# Hints to Young Practitioners—No. IV

**Published:** 1872-10

**Authors:** 


### HINTS TO YOUNG PRACTITIONERS—No. IV.

BY SENEX.

If afflicted humanity were restricted to one remedy for its ailments, beyond all doubt, the general choice, perhaps without a dissenting voice, would be opium. The ancient Egyptians deified the leek; if the days of idolatry should return, the poppy would surely be worshipped.

After Iago had poured the poison of jealousy into the soul of the noble Moor, the great bard makes the villain exclaim: "Not poppy, nor mandragora, nor all the drowsy syrups of the world, shall medicine to thee that sweet sleep which thou ow'dst yesterday."

Mandragora, like a hundred other drugs of the day (a similar fate probably awaits many articles of our Dispensatory), has passed away, but time only elevates the magnitude of the boon conferred on man by a merciful Providence in the gift of opium. Its hypnotic and pain-relieving properties are matters of daily and hourly experience. Half a century ago, Dr. Linn called the attention of the profession to its value in fever. We all know that fever, with the exception of the intermittent type (curable by quinine), has a destined course to run, determined by pathological laws not yet understood. We appeal to the great depurating emunctories, with our emetics, purgatives, diaphoretics and diuretics, and we endeavor to sustain the general powers with food and wine—thus keeping our patient alive until certain results are worked out, more perhaps by the vis medicatrix naturæ, than by our drugs. Amongst the helps which we think we render, and undoubtedly do render to nature, none holds a higher rank than opium, judiciously administered. A third of a grain of morphia will often bring almost heavenly rest to the sufferer from fever, and it will generally induce perspiration. There need be no backwardness in its use on account of a high state of fever, nor even of delirium. After a hot mustard pediluvium, with ice water to the forehead, a good dose of morphine will calm ordinary delirium. As a tranquilizer of the nervous system, and sedative to exalted arterial action, it is, in my judgment, far more valuable than aconite or veratrum viride. It seems to me that these are often given on unsouud therapeutic principles. It is very questionable whether we are any nearer the crisis or termination of a fever or a pneumonia, with the heart's strokes brought down ten or twenty beats in the minute, than before. The subtle, systemic poison is at work just the same. But morphine usually brings comfort, and though it may not shorten the duration of an illness, helps wonderfully in smoothing the passage through it. In the phlegmasia accompanied by pain, it is our sheet-anchor. In pleurisy and peritonitis, it has no rival.

The hypodermic syringe is a most valuable invention, enabling us to mke sure of the introduction of medicines when the stomach is incapable of receiving them; one should always be carried in the pocket.

The power of opium to allay spasm is sometimes manifested in a striking manner. The late Professor Wellford, of Richmond, attended, in consultation with the writer, a case of shoulder presentation in a primiparous young woman. The hand of the child was hanging from the vulva; the waters were drained off; the throes tremendous, wedging the body in the pelvis more and more. All our efforts failing, we opened a vein, bled to forty ounces, and gave four grains of opium. At our meeting in an hour afterward, we were gratified to find the child born by spontaneous evolution, the head coming down,

Another eminent Virginia physician, Dr. George F. Carmichael, met the writer in consultation in a case of strangulated inguinal hernia. The taxis failing, we ordered a hot bath and four grains of opium. At our meeting soon after, we had the satisfaction to find our patient spontaneously relieved.

It is true these are exceptional cases, but it is no small thing to have such weapons of war in our therapeutic armory.

Combined with calomel, in the proportion of two grains of calomel, and one-sixth of a grain of opium, we have a medicine of peculiar power in some of the conditions of fever, with or without local affection. Your patient has been already purged, but the tongue keeps loaded; there is no diminution of fever; perhaps there is great restlessness, and it may be, with a sense of oppression about the epigastrium. You may or may not give an emetic of ipecac, which often brings about a good result, and then you put your patient on calomel and opium, giving one of the powders every two hours, to six powders, and following them with a dose of castor oil.

Combined with quinine, morphine has an added power in severe neuralgic pain. Ten or fifteen grains of quinine, with half or three-fourths of a grain of morphine, will hardly fail of bringing relief.

In those terrible attacks known as nephritic colic, how powerless would the physician be without chloroform and morphia! And what would the unhappy victim of cancer do without opium? Where we can not cure, it is often given us to smooth the inevitable passage to the grave, and to effect a comparatively easy and tranquil euthanasia. Let us thank God for opium!

SAVANNAH, GEORGIA, October 19th, 1872.

ERRATA.—In the last article by "Senex," published in the September number, page 367, third line of the article, for "ten grains of calomel," read *two*.

---

A useful form of disinfectant has been brought into use in England—namely, saw-dust soaked in a saturated solution of carbolic acid. It is convenient for many purposes, cheap, easily prepared, and not liable to be swallowed accidentally, as ordinary liquid disinfectants are.—*Med. and Surg. Journal, Boston.*

